# Mode and specificity of binding of the small molecule GANT61 to GLI determines inhibition of GLI-DNA binding

**DOI:** 10.18632/oncotarget.2046

**Published:** 2014-05-31

**Authors:** Akwasi Agyeman, Babal K Jha, Tapati Mazumdar, Janet A Houghton

**Affiliations:** ^1^ Department of Cancer Biology, Lerner Research Institute, Cleveland Clinic, Cleveland, OH

**Keywords:** GANT61, GLI, binding

## Abstract

The GLI genes, GLI1 and GLI2, are transcription factors that regulate target genes at the distal end of the canonical Hedgehog (HH) signaling pathway (SHH->PTCH->SMO->GLI), tightly regulated in embryonic development, tissue patterning and differentiation. Both GLI1 and GLI2 are oncogenes, constitutively activated in many types of human cancers. In colon cancer cells oncogenic KRAS-GLI signaling circumvents the HH-SMO-GLI axis to channel through and activate GLI in the transcriptional regulation of target genes. We have observed extensive cell death in a panel of 7 human colon carcinoma cell lines using the small molecule GLI inhibitor GANT61. Using computational docking and experimental confirmation by Surface Plasmon Resonance, GANT61 binds to the 5-zinc finger GLI1 protein between zinc fingers 2 and 3 at sites E119 and E167, independent of the GLI-DNA binding region, and conserved between GLI1 and GLI2. GANT61 does not bind to other zinc finger transcription factors (KLF4, TFIIβ). Mutating the predicted GANT61 binding sites in GLI1 significantly inhibits GANT61-GLI binding and GLI-luciferase activity. Data establish the specificity of GANT61 for targeting GLI, and substantiate the critical role of GLI in cancer cell survival. Thus, targeting GLI in cancer therapeutics may be of high impact.

## INTRODUCTION

Hedgehog (HH) signaling plays a critical role in normal cellular processes. It is pivotal in embryogenesis, tissue patterning, and differentiation [1-3]. The canonical HH pathway is critical to normal mammalian gastrointestinal development, where it is involved in the coordinate regulation of differentiation of normal intestinal villi [4-6]. The GLI genes, GLI1 and GLI2, are transcription factors that regulate target genes at the distal end of the canonical HH pathway (SHH->PTCH->SMO->GLI). Their expression in these processes is tightly regulated [1-3], with little expression detected in adult tissues [7]. GLI1 and GLI2 are transcriptional activators, binding to GACCACCCA-like consensus promoter sequences [1, 8, 9]. From genetic and biochemical studies, we and others suggest that GLI2 is the primary mediator of HH signaling, which activates GLI1 to transcriptionally regulate target genes and augment HH signaling quantitatively as well as qualitatively [1, 9-11]. Differences in the biological activities of GLI1 and GLI2 are evident, since GLI1^−/−^ mice have no obvious phenotype [11], in contrast to homozygous GLI2^−/−^ mice which die at birth [12, 13]. During development, GLI1 is strongly expressed along the midline and is a marker of the response to SHH. In contrast, GLI2 is expressed in the lateral regions, suggesting regulation by alternate factors [14]. GLI1 and GLI2 possess both independent and overlapping functions [1, 9-12, 15].

Both GLI1 and GLI2 are oncogenes, induce transformation and tumorigenesis [16-18], and are constitutively activated in many types of human cancers [1, 15]. Failure to terminate HH/GLI signaling, which occurs in cancer, leads to an amplified and persistent increase in GLI1 and GLI2 activity (reviewed in [15]). Amplification of GLI1 or GLI2, mutations in PTCH or SMO, upregulated expression of HH ligands, and activating mutations that initiate transformation can dysregulate HH signaling [1, 15]. Small molecule inhibitors of SMO upstream of GLI have probed the canonical, HH-SMO-GLI axis in preclinical models [19-25] and in human cancers [24, 26-28]. SMO inhibitors have limited or no clinical activity (GDC-0449, IPI-926, LDE225; reviewed in [24, 26]), except in a small number of HH-GLI-dependent tumors (e.g. basal cell carcinoma [29, 30], medulloblastoma [26, 31]). Acquired resistance to SMO antagonists also occurs [32].

**Figure 1:**
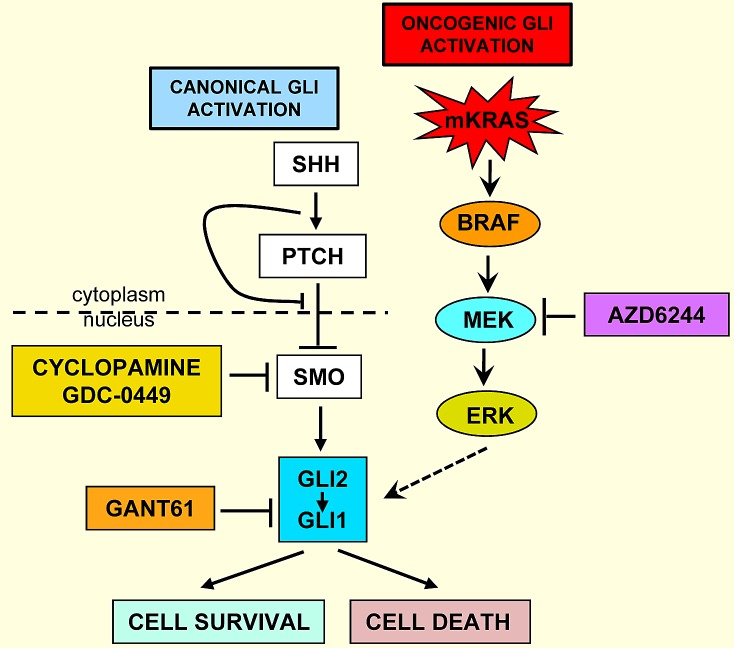
Schema of pathways for the aberrant activation of GLI in colon cancer

Constitutive GLI activation progresses during colon carcinogenesis and in metastatic disease [21, 33, 34] by ligand-dependent (canonical) and ligand–independent (oncogenic) mechanisms [35-39]. Oncogenic pathways (KRAS/BRAF in colon cancer) circumvent the canonical HH-GLI axis by converging on and further driving GLI to a higher activating state in tumor cells, promoting cellular proliferation, tumor progression and survival [8, 15, 19, 40-42 43, 44]. Thus, potential targets upstream of GLI are bypassed, including SMO. Activating mutations in both KRAS [15, 42, 45-49] and BRAF [19, 48, 50, 51] are prevalent, and occur in high frequency in colon cancers [47-49, 51-53]. We have demonstrated that oncogenic KRAS/BRAF signaling activates GLI independent of the HH-SMO-GLI axis [38], inhibited by pharmacologic inhibitors of MEK (U0126 [38], AZD6244), and by GANT61, which targets GLI and GLI-dependent transcription. We have demonstrated that MEK inhibitors reduce GLI-luciferase activity [38]. Thus, GANT61 is effective when GLI (GLI1+GLI2) serves as a common node of activation through which oncogenic signals converge (schema, Figure 1). Due to the extensive cytotoxicity induced by GANT61 in human models of colon cancer [36, 38, 39], data suggest that GLI is a critical target in colon cancer cell survival, and also in other cancers where GLI is constitutively activated and/or an oncogenic KRAS-GLI axis drives proliferation.

GANT61, an experimental agent in preclinical studies, was originally identified in a cell-based screen for small molecule inhibitors of GLI1-mediated transcription [54]. In this study GANT61 abrogated GLI function in the nucleus, blocked both GLI1- and GLI2- mediated transcription, inhibited GLI1-DNA binding, and demonstrated anti-tumor activity against human prostate cancer xenografts. We have demonstrated rapid inhibition of GLI1 and GLI2 binding to target gene promoters (1 hr), reduced reporter activity specific to GLI-luciferase, and rapid inhibition of gene transcription in human colon carcinoma cell lines [37]. Overexpression of GLI1 or GLI2 also protects cells from GANT61-mediated cell death [39]. Due to our findings of the critical role of GLI in colon cancer cell survival, and the importance of GANT61 as a unique small molecule inhibitor, we sought to determine the mechanism and specificity of GANT61 binding activity. We investigated whether GANT61 binds to the GLI protein or to DNA sequences, and further determined the exact sites of interaction.

GLI1 and GLI2 are zinc finger proteins, one of the most common DNA-binding motifs in eukaryotic transcription factors [7, 55]. The crystal structure of the five zinc finger GLI1-DNA complex is known (PDB ID 2GLI) [55]. Fingers 2 through 5 of GLI1 bind in the major groove and wrap around the DNA, with fingers 4 and 5 making the most extensive base contacts in a conserved 9-bp region. Fingers 2 and 3 only make a single base contact. Finger 1, which does not contact the DNA, makes extensive protein-protein interactions with Finger 2 [55]. To gain insight into the structural binding characteristics of GANT61, our findings from computational docking of GANT61 to PDB ID 2GLI, and experimentally by Surface Plasmon Resonance (SPR) technology, determined that docking of GANT61 to both the GLI1 protein and to the GLI1-DNA complex was identical; GANT61 did not bind to DNA or to other zinc finger transcription factors. The K_D_ for dissociation (3.2-7.5 μM) and GANT61 concentrations that induce cell death in intact cells (10-20 μM) are in the same low μM range. These studies critically evaluate the mode of binding of GANT61, and identify that GANT61 specifically binds to GLI1 protein, which is strongly dependent on 2 amino acids. Overall these findings substantiate the importance of GLI as a target in cancers with activated GLI and/or oncogenic KRAS/BRAF signaling, and that inhibition of GLI-dependent transcription by a specific small molecule inhibitor can have profound effects on cell survival.

## RESULTS

### Critical role of GLI in cell survival

**Figure 2:**
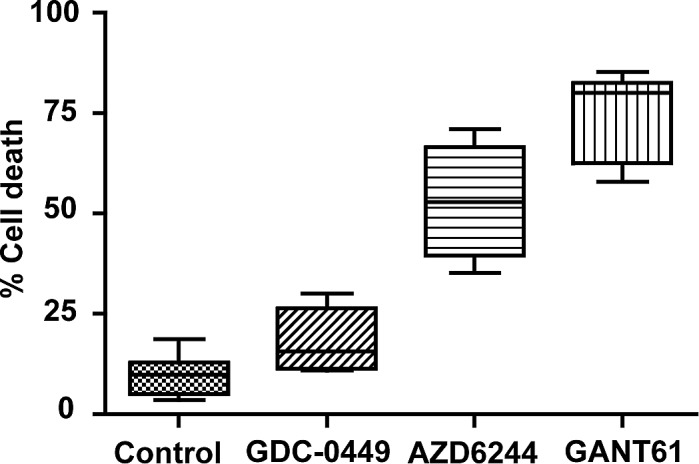
Human colon carcinoma cell lines were treated for 72 hr, in duplicate, to equimolar concentrations (20 μM) of GDC-0449 (n=5), AZD6244 (n=4), GANT61 (n=7), or were untreated (n=7). Cells were harvested by trypsinization, cell death analyzed by Annexin V/PI staining and FACS analysis, and raw data quantitated using CellQuest software, as described in Materials and Methods. Data represent the Mean, Range, and SD.

Following 72 hr drug exposure with equimolar concentrations of drugs (20 μM), inhibition of SMO in human colon carcinoma cell lines by GDC-0449 induced minimal cell death [36, 38, 39], while cell lines (HT29, SW480, HCT116, GC3/c1) with aberrant KRAS/BRAF signaling [51] demonstrated sensitivity to the MEK inhibitor AZD6244. GANT61 induced extensive cell death following termination of oncogenic signaling at the level of GLI in all of the 7 cell lines (Figure 2).

### Computational docking of GANT61 to GLI1 or DNA

**Figure 3:**
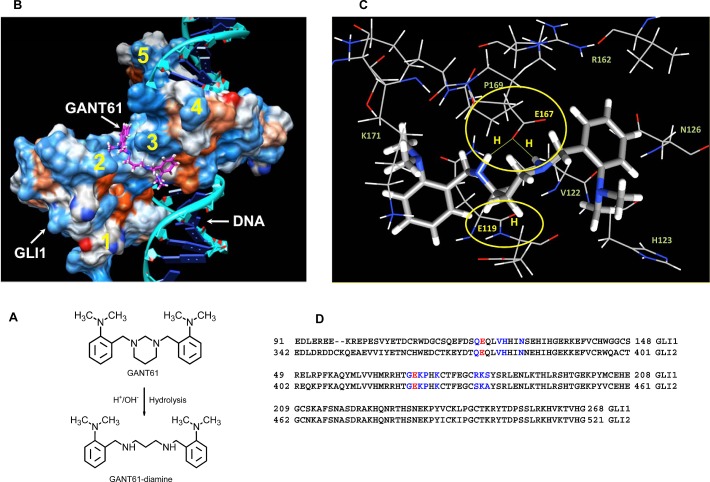
Computational docking of GANT61 to GLI1 using the known crystal structure of the five zinc finger GLI1-DNA complex (PDB ID 2GLI) [55]. A: Two dimensional chemical structures of GANT61 and GANT61-diamine drawn in ACD-ChemSketch (Advanced Chemistry Inc.); B: Computational modeling of GANT61-diamine binding predicts binding to GLI1 within the groove between fingers 2 and 3; zinc fingers 1-5 (yellow); C: Predicted GANT61-diamine bound to GLI1 at amino acids E119 (1 H bond) and E167 (2 H bonds), amino acids residues within 3.5°A of any atom of GANT61 are shown as line. D: GLI1 and GLI2 alignment; E119, E167 (red); residues within 3.5 Å of docked GANT61-diamine (blue).

Computational docking analysis of GANT61 to GLI1, DNA, or the GLI1-DNA complex utilized GANT61-diamine, the active form of GANT61, in the analysis (Figure 3A). Docking predicted that GANT61-diamine binds to the GLI1 protein, but not to the DNA binding site, at amino acids E119 and E167, which lie within the groove between zinc fingers 2 and 3, on the opposite surface from, but in close proximity to, the GLI1-DNA binding region (Figure 3B). Predicted docking of GANT61-diamine is via two-way H bonds at E167 (2.2Å) involving the two imino protons of GANT61-diamine, and one H bond at E119 involving one imino proton (Figure 3C). No significant change in GANT61-diamine docking was observed when a complex of GLI1-DNA was used as the target; GANT61-diamine was not observed to bind with DNA. Alignment of GLI1 and GLI2 amino acid sequences, using Clustal 2.1, demonstrated that the residues within 3.5 Å of GANT61-diamine ligand atoms are conserved (Figure 3D). Computational modeling predicts the biological activity of GANT61 to be mediated by a direct GANT61-GLI1 interaction.

### GANT61 inhibits GLI1-DNA interactions by binding to GLI1 and not to DNA

**Figure 4:**
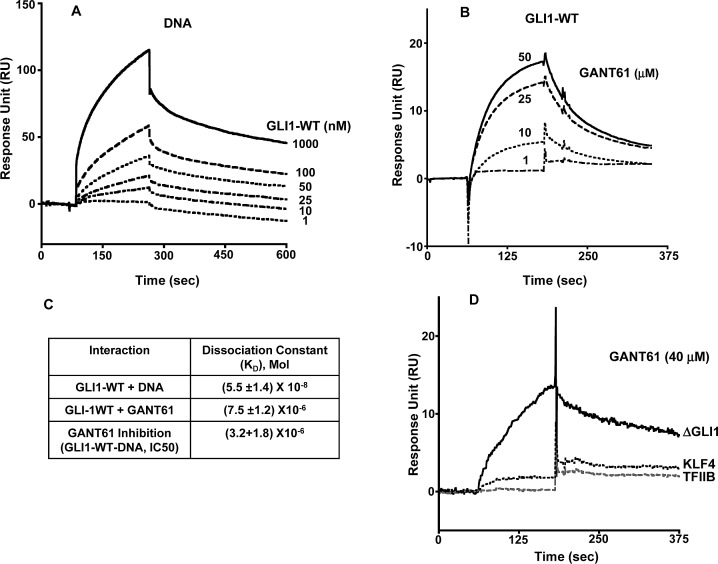
Surface Plasmon Resonance (SPR) of: A: Immobilized DNA with increasing concentrations of GLI1-WT as analyte: B: Immobilized GLI1-WT with determination of GANT61 binding in presence of increasing concentrations of GANT61 (1-50 μM); C: Dissociation Constants (K_D_) for the interaction between GLI1-WT and DNA, GLI1-WT and GANT61, or inhibition of GLI1-WT-DNA binding by GANT61; D: Binding of GANT61 (40 μM) to immobilized ΔGLI-WT in contrast to no binding of GANT61 (40 μM) to immobilized KLF4 or TFIIβ. The analyte was exposed to sensor chips for 3 min followed by 5 min in the absence of analyte as described in Materials and Methods. Response Units are shown vs time of incubation (sec).

**Figure 5:**
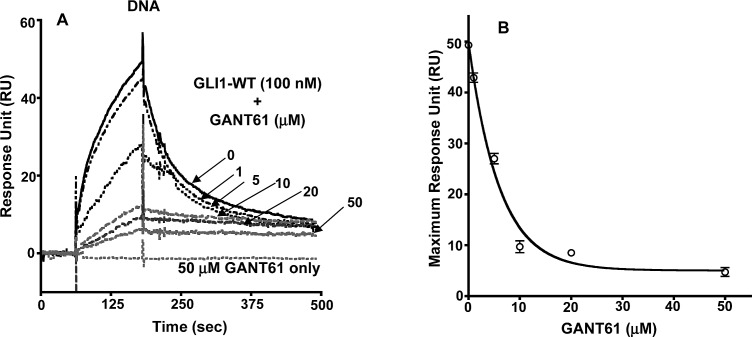
SPR of: A: Immobilized DNA +/− GLI1 (100 nM) +/− GANT61 at varied concentrations (1-50 μM); B: Maximum RU vs concentration of GANT61.

To critically evaluate the structural characteristics of the GANT61-GLI-DNA binding interactions, we utilized SPR technology. To confirm the GLI-DNA interactions, biotinylated DNA (100 nM) was immobilized on a streptavidin pre-coated SA sensor chip (GE Healthcare) with varying concentrations of GLI1 (1–1,000 nM) as the analyte. Full length GLI1 binds to immobilized DNA on the sensor chip in a dose dependent manner (Figure 4A); the dissociation constant for binding (K_D_) was 5.5 μM (Figure 4C). To determine whether GANT61 binds directly to GLI1 as predicted from the docking model, GLI1 was immobilized on a CM5 sensor chip with varying concentrations of GANT61 (1–50 μM) as the analyte. A dose-dependent increase in the resonance response was observed (Figure 4B). Significant binding was determined at 10 μM GANT61 and was close to maximum at 25 μM GANT61. The K_D_ for GANT61-GLI1 binding was 7.5 μM. To determine whether the binding of GANT61 was specific to GLI1 and not to other zinc finger transcription factors, KLF4 (Kruppel-like factor) or TFIIβ (general transcription factor) were each immobilized to sensor chips, and a supraphysiological concentration of GANT61 (40 μM) used as the analyte. Binding was compared to GANT61-ΔGLI-WT binding. No significant binding of GANT61 to KLF4 or TFIIβ was detected (Figure 4D). To investigate whether GANT61 is able to disrupt the DNA-GLI1 interaction, and also to determine whether GANT61 binds to DNA, DNA (100 nM) was immobilized, and GANT61 (50 μM) in the absence or presence of GLI1 (100 nM) was employed as the analyte. In the absence of GLI1, there was no detectable binding of GANT61 to DNA. In contrast, in the absence of GANT61, significant binding of GLI1 to DNA was determined (Figure 5A). Subsequently, increasing concentrations of GANT61 (1–50 μM) were employed as the analyte in the presence of a fixed quantity of GLI1 (100 nM). A significant decrease in the binding of GLI1 to DNA was determined with GANT61 (5 μM), and close to maximal inhibition of binding at 10 μM GANT61 (Figure 5A). Maximum RU in the presence of GLI1 demonstrated a concentration-dependent decrease as the GANT61 concentration was increased and GLI1-DNA binding was inhibited (Figure 5B). The K_D_ for GANT61 inhibition of GLI1-DNA binding was 3.2 μM (Figure 4C).

### E119A and E167A mutations reduce GANT61 binding to GLI1

**Figure 6:**
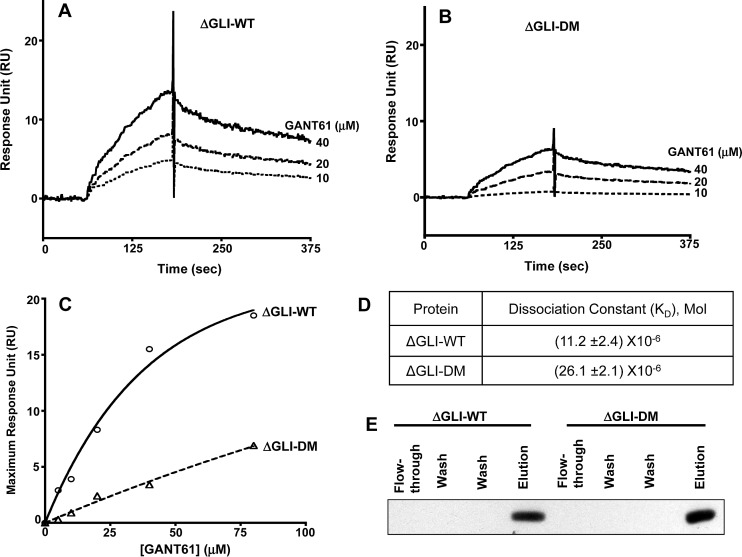
SPR conducted with immobilized ΔGLI-WT or ΔGLI-DM proteins following site directed mutagenesis of GLI1-WT, PCR amplification and protein purification of ΔGLI-WT without mutation, or ΔGLI-DM with both E119 and E167 sites mutated. Immobilized A: ΔGLI-WT or B: ΔGLI-DM with varied concentrations of GANT61 (10-40 μM) as analyte; C: Maximum Response for binding of GANT61 to ΔGLI-WT or ΔGLI-DM at varied concentrations of GANT61. D: Dissociation Constants (K_D_) for binding of GANT61 to ΔGLI-WT or ΔGLI-DM proteins; E: Purification of ΔGLI-WT or ΔGLI-DM proteins on Ni-NTA columns as described in Materials and Methods.

To determine the functional significance of the putative sites (E119, E167) on GLI1 in the GANT61-GLI1 interactions, fragments containing E119A and E167A mutations were amplified by PCR, cloned, expressed and purified using Ni-NTA affinity chromatography, as described in Materials and Methods. Purified proteins were immobilized on Ni-NTA sensor chips, and GANT61 (10-40 μM) was used as the analyte (ΔGLI-WT, Figure 6A; ΔGLI-DM, Figure 6B). Significant inhibition of GANT61-GLI binding was determined following mutation of the two predicted binding sites, indicating the critical importance of these sites in the small molecule-protein interaction. Upon further analysis of a comparison of RU^Max^ values (Figure 6C), mutagenesis of the two binding sites inhibited GANT61-GLI binding by ≈ 60%, yielding K_D_ values for ΔGLI-WT and ΔGLI-DM of 11.2 μM and 26.1 μM, respectively (Figure 6D).

### GLI-luciferase activity

**Figure 7:**
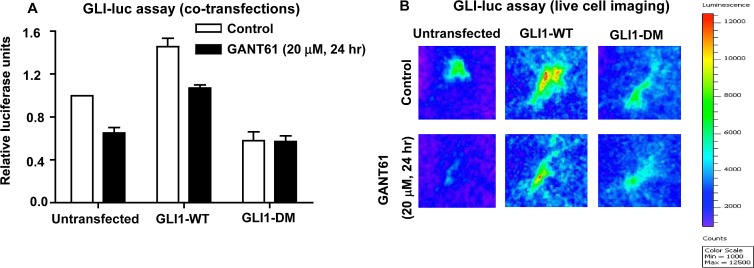
GLI-luciferase reporter assays in HT29 cells under 2 different conditions: A: Co-transfection of GLI-luc, pRLTK, and either pBabe-Puro, GLI1-WT or GLI1-DM cDNA into HT29 cells using Lipofectamine 2000; 24 hr post-transfection, cells were treated with GANT61 (20 μM) for 24 hr, and luciferase activity determined as described in Materials and Methods. B: An HT29-derived stable cell line expressing GLI-luc was transfected with either pBabe-Puro, GLI1-WT or GLI1-DM cDNA, and exposed to GANT61 (20 μM) for 24 hr. Live cell imaging was performed using a Bruker optical and X-ray small animal imaging system, as described in Materials and Methods.

To evaluate the biological significance of the mutations introduced in the GLI1 protein (E119A, E167A), the effect of full length GLI1-DM on GLI-luc reporter activity was determined in HT29 cells. The cells were transiently cotransfected with pBabe-Puro, GLI1-WT cDNA, or GLI1-DM cDNA and both GLI-luc and pRLTK. Cells expressing GLI1-WT demonstrated a 7-fold increase in GLI-luc activity within 24 hr of transfection; in contrast, GLI1-DM increased GLI-luc activity by only 2-fold. Further, cells expressing GLI1-DM did not demonstrate decreased GLI-luc activity while cells expressing GLI1-WT demonstrated reduced GLI-luc activity in response to GANT61 (20 μM, 24 hr; Figure 7A). To further corroborate the biological effect of the GLI1-DM mutant, we employed an HT29-derived stable cell line that constitutively expresses GLI-luc. HT29-GLI-luc cells were transfected with pBabe-Puro, GLI1-WT or GLI1-DM plasmids and luciferase activity was visualized by live cell imaging. Similar to the findings with co-transfection, cells expressing GLI1-WT demonstrated increased GLI-luc activity while cells expressing GLI1-DM demonstrated reduced GLI-luc activity (Figure 7B). Administration of GANT61 (20 μM, 24 hr) reduced GLI-luc activity in both the untransfected and GLI1-WT-expressing cells but not in cells expressing GLI1-DM, which were insensitive (Figure 7B). Collectively, data demonstrate that mutating both GANT61 binding sites in the full length GLI1 cDNA significantly inhibits GLI1-DNA binding activity, resulting in reduced GLI-luc reporter activity, in addition to rendering resistance to GANT61.

## DISCUSSION

GLI is constitutively activated in a wide variety of human cancers, and we have demonstrated the critical role of GLI as a target in colon cancer cell survival using GANT61 as a probe [35-39]. In contrast we have demonstrated minimal effects on GLI targets in human colon carcinoma cells in response to physiologically relevant concentrations of SMO inhibitors [37-39]. Cells respond at higher concentrations of GDC-0449 (100 μM), used to select for drug resistance, and these cells remain sensitive to GANT61 [39]. The relative inactivity of SMO inhibitors in this model and others except in certain HH-GLI-dependent tumors [19-28], underscores the importance of activation of GLI by non-canonical pathways that drive cell proliferation and survival, in particular oncogenic KRAS/BRAF. We have shown decreased p-Erk and reduced expression of GLI targets and GLI-luciferase activity in human colon carcinoma cells following exposure to MEK inhibitors [38]. Cell line models with an activated KRAS pathway are found to be more sensitive to inhibition of MEK [56]. GLI therefore serves as a point of convergence and nodal activation by oncogenic signaling pathways, made evident by the extensive cytotoxicity induced by the small molecule inhibitor of GLI transcription, GANT61. However little is known regarding the mechanism by which GANT61 inhibits transcriptional activity of the GLI proteins.

The GLI genes are members of the GLI-Kruppel family of transcription factors [7, 55, 57]. Analysis of the GLI gene sequence indicates that it encodes a 118 kd protein that contains five repeats of a zinc finger DNA-binding motif. From the crystal structure of the 5-zinc finger GLI-DNA complex, it has been determined that finger 1 does not contact the DNA, while fingers 2-5 bind in the major groove and wrap around the DNA. Zinc fingers 4 and 5 of GLI1 make extensive base contacts in a conserved 9-base pair region [55]. Using this crystal structure of the GLI1-DNA complex, predicted docking of GANT61 to the GLI1 protein, the GLI1-DNA complex or to DNA itself, was determined. The predictions were that GANT61 binds between zinc fingers 2 and 3 of the GLI1 protein, at amino acids E119 and E167 by H bonds, in close proximity to, but independent of the DNA binding region. Close inspection revealed that the molecular surface where GANT61 binds is opposite from the groove where DNA binds. The sequence alignment showed that most of the residues within 3.5 Å of GANT61 are conserved between GLI1 and GLI2. BIAcore instrumentation using SPR-based biosensors has made it possible to routinely study the binding of small molecules (< 500 kD) to targets [58], and has been successfully employed to directly measure and quantitate the binding of small molecule inhibitors to target proteins, e.g. the p38-mitogen-activated protein kinase (MAPK) [59]. First, binding of GLI1 to immobilized DNA, or binding of GANT61 to immobilized GLI1, were demonstrated to be dependent on the concentration of GLI1 or GANT61, respectively, as the analyte. Second, the binding of GLI1 to DNA was inhibited by GANT61. No significant binding of GANT61 to DNA was determined even at the highest non-physiologic concentration (50 μM) of GANT61 employed. These data confirm the specificity of binding of GANT61 to the GLI1 protein, and negate any direct effect of GANT61 on binding to DNA. They also demonstrate inhibition of the binding of new GLI1 to DNA. We have demonstrated the appearance of γH2AX foci within 4 hr of exposure of cells to GANT61 [38, 39], which we now know to be independent of direct DNA binding. This is in contrast to other agents that induce DNA damage by direct DNA binding, including cisplatin, doxorubicin or etoposide. Our data are consistent with the introduction of DNA strand breaks and DNA damage due to inhibition of transcription, which has been shown to create R-loops (RNA:DNA hybrids) at sites of stalled RNA polymerase II, and has been associated with the mechanism of action of camptothecin. Thus, stabilized TopI cleavable complexes induced by camptothecin are potent transcription-blocking DNA lesions during elongation [60, 61]. GANT61-induced DNA damage is recognized at the initiation of S-phase with induction of a transient intra-S-phase checkpoint, where cells accumulate in early S, fail to progress, and undergo cell death [37-39]. Thus, DNA damage occurs prior to the induction of apoptosis. These mechanisms are currently being explored.

The specificity of GANT61-GLI1 interactions were further evaluated by using 2 other zinc finger proteins/transcription factors, KLF4 and TFIIβ: KLF4 is a member of the GLI-Kruppel family with the classic Cys_2_His_2_ zinc finger structure [57, 62]; the general transcription factor TFIIβ, which has a zinc ribbon fold characterized by two beta-hairpins that form two structurally similar zinc-binding sub-sites [63]. GANT61 did not bind to KLF4 or TFIIβ, suggesting a binding mode unique to GLI1. Of interest, the predicted binding constants (K_D_) for GANT61-diamine-GLI1 (7.5 μM) or GANT61 inhibition of GLI-DNA binding (3.2 μM) are consistent with GANT61 concentrations of 10 μM-20 μM for 48 hr-72 hr exposures, required to induce cell death in human colon carcinoma cell lines [36-38].

To determine the importance of the predicted binding sites for GANT61 at E119 and E167 in the GLI1 protein, site-directed mutagenesis generated a double mutant (DM) with each of the two binding sites converted from E -> A. Following immobilization of the wild type or double mutant proteins on CM5 sensor chips and using GANT61 as the analyte, binding of GANT61 to ΔGLI-DM was decreased by 60%, with the K_D_ for dissociation increased by 2.3-fold. This translated into inhibition of the ability of GANT61 to decrease GLI-luciferase activity in cells expressing GLI1-DM, in contrast to the inhibition of GLI-luc activity in untransfected cells or in cells transfected with GLI1-WT.

In summary, we have demonstrated that GLI1 is a specific target for the small molecule inhibitor, GANT61, which binds directly to the GLI1 protein and not to the DNA or to other zinc finger transcription factors. This provides a unique mechanism of induction of DNA damage following inhibition of GLI-dependent transcription, not related to direct DNA binding. These data underscore the critical importance of GLI as a target in survival of cancer cells. GLI1 and GLI2 are constitutively activated in colon cancer cells by oncogenic signaling pathways upstream of GLI. Targeting GLI terminates HH-SMO-GLI, KRAS-GLI, and HH-signaling in these cells, and is a more effective targeting strategy than employing agents that function upstream in these pathways. Colorectal cancer is the second leading cause of cancer deaths in the United States with >150,000 new cases annually [64, 65], and remains refractory to standard treatment approaches. Further, GLI1 and/or GLI2 are oncogenes, and are constitutively activated in many types of human cancers including epithelial cancers of the GI tract, brain tumors, melanoma, pediatric solid tumors, liver, lung, breast, pancreatic and prostate cancers. KRAS is mutated in 30% of all human cancers, and in 50% of colon carcinomas. Targeting GLI for therapeutics therefore has the potential for high impact.

## MATERIALS AND METHODS

### Cell Culture

Human colon carcinoma cell lines HT29, SW480, HCT116, GC3/c1, VRC5/c1, HCT8, RKO, have been described previously [35-38, 51]. All cell lines including HT29 were cultured in 10% FBS-supplemented RPMI medium and maintained at 37°C with 5% CO_2_.

### Analysis of cell death

Human colon carcinoma cell lines were treated, in duplicate, with equimolar concentrations (20 μM) of each agent that inhibits SMO (GDC-0449; JS Research), MEK (AZD6244; Selleckchem) or GLI (GANT61; Calbiochem). Following 72 hr exposure, cells were collected by trypsinization and incubated with Annexin V FITC (BD Biosciences, CA) and propidium iodide (Sigma, MO) prior to analysis using a FACSCalibur flow cytometer. Raw data were analyzed by CellQuest software [36-39].

### Molecular Docking

Using the known crystal structure of the five zinc finger GLI1-DNA complex (PDB ID 2GLI) [55], a prediction of how GANT61 may bind to GLI1, to DNA, or to the GLI1-DNA complex, was obtained. An unbiased docking of GANT61 was performed using AutoDock 3.0.5 with Autodock tools (ADT), as described [66]. The docking procedure allows GANT61 flexibility and the exploration of a large number of binding modes. GANT61 is a hexahydropyrimidine that hyrolyzes in solution at all pHs to an active diamine, which is the biologically active form of GANT61 [67]. The two-dimensional structure of GANT61-diamine was obtained from Lauth et al [67] and converted into the three-dimensional structure by using Accelrys Discovery Studio 1.7; energy minimization was achieved using CHARM (Accelrys Inc). The file for GLI1 was downloaded from the protein data bank (www.rcsb.org) and imported to ADT, adding polar hydrogen, charge, and solvation parameters; data for GANT61-diamine were also imported to ADT. For initial docking, the grid volume was set to accommodate the entire GLI1 molecule, with refinement performed using a grid volume consistent with the molar volume of GANT61 with spacing of 0.4 Å. A composite file of all possible conformations of GANT61-diamine with GLI1, DNA, or the GLI1-DNA complex was compiled. All three-dimensional docked complexes were analyzed and visualized by UCSF Chimera [68] and pymol (www.pymol.com).

### Site Directed Mutagenesis of GLI1

Full length GLI1 cDNA was a gift from Dr. Graham Neill, Queen Mary University of London, UK. Two complimentary oligonucleotides for the double mutation (A50C/A65C) to convert E -> A at amino acids E119 and E167, were purchased from Integrated DNA Technology (Coralville, IA). The mutation reaction was set up using the QuikChange II Site-Directed Mutagenesis kit (Agilent Technologies Inc.). The reaction mixture contained 50 ng of template DNA (pBabe-GLI1), 125 ng each of the oligonucleotide primer, 200 μM dNTP mix, 2.5 U of PfuUltra HF DNA polymerase and I × reaction buffer in a total volume of 50 μl. The samples were denatured at 95°C for 30 sec and then cycled 16 times at 95°C for 30 sec., 55°C for 1 min followed by 68°C for 9 min. The parental plasmid DNA was digested by adding 10 U of Dpn I restriction enzyme to the amplification reaction and incubated at 37°C for 1 hr. DH5α competent cells (Agilent Technologies) were transformed with 1 μl Dpn I-treated DNA and plated on LB-agar plate containing 100μg/mL ampicillin. Plates were incubated at 37°C for 16 hr. Single colonies were isolated and plasmid DNA extracted and sequenced for mutation verification.

### Sub-cloning of ΔGLI-WT and ΔGLI-DM mutant (A50C/A65C) fragments

Based on 1) the published crystal structure of the GLI1-DNA complex (PDB ID 2GLI) [55], and 2) the molecular modeling of GANT61-GLI1 binding, the GLI1 Zn-finger domain with DNA binding sequence was amplified by PCR to generate ΔGLI-WT and ΔGLI-DM fragments, which were subcloned into BamH1 and SalI sites of the pHIS-II-1 plasmid using the Infusion cloning kit (Clontech). The reaction mixture contained 1x infusion HD enzyme premix, 40 ng of pHISII-1 [69] linearized with BamH1 and Sal1 and 50 ng of ΔGLI-WT or ΔGLI-DM fragment in a total volume of 10 μl. The reaction was incubated at 50°C for 15 min and then placed on ice. DH5α competent cells were transformed with 2 μl of the cloning reaction and plated on LB-agar plate with 100 μg/ml ampicillin. The plates were incubated at 37°C overnight and single colonies were grown in a 5 ml culture. Plasmid DNA was extracted and sequenced for verification of the presence of the ΔGLI-WT or ΔGLI-DM fragment.

### Protein Expression and purification

BL21(DE3) bacterial cells (Agilent Technologies) were transformed with either pHIS-II-1-ΔGLI-WT or pHIS-II-1-ΔGLI-DM. Single colonies were grown at 37°C and shaking at 225 rpm overnight in 5 ml LB medium containing 100 μg/ml ampicillin. The culture was diluted 1:10 and shaken at 37°C at 225 rpm until the optical density at 600 nm reached 0.7. The culture was cooled to 4°C and IPTG was added to a final concentration of 0.3 mM. The culture was then grown at 20°C for 18 hr. Cells were harvested and washed with 50 mM Tris pH 8.5 buffer containing 150 mM NaCl. Cell pellets were resuspended in 10 ml of lysis buffer (50 mM Tris, pH 8.5, 5 mM MgCl_2_, 50 mM KCl, I mM EDTA, 5% glycerol, 14 mM 2-mercaptoethanol, 50 μM PMSF) containing 1x protease inhibitor cocktail (Roche). The cells were sonicated for 15 cycles (10 sec ‘on’ and 15 sec ‘off’, each cycle). The sonicated fraction was centrifuged and the supernatant was bound to Ni-NTA. The Ni-NTA-bound protein was washed × 3 with washing buffer (50 mM Tris, pH 8.5, 5 mM MgCl_2_, 500 mM NaCl, I mM EDTA, 5% glycerol, 7 mM 2-mercaptoethanol, 50 μM PMSF) followed by washing × 2 with washing buffer containing 10 mM imidazole. ΔGLI-WT and ΔGLI-DM proteins were eluted with wash buffer containing 200 mM imidazole, and detected by SDS-PAGE/coomassie blue staining and by Western analysis using the His-probe antibody H15 (Santa Cruz Biotechnology).

### Surface Plasmon Resonance

The full length GLI1 protein was purchased from OriGene, KLF4 from Peprotech, and TFIIβ from Abcam. Amine group covalent coupling chemistry was used to immobilize GLI1, KLF4 or TFIIβ proteins on a CM5 sensor chip (GE Health Care) via free primary amine groups (lysine residues), which are present in GLI1, and also present in KLF4 and TFIIβ. The analyte was GANT61. To perform the reverse binding, the biotin-labeled synthetic GLI1 DNA-binding sequence (21-mer; purchased from Integrated DNA Technologies [IDT]), was captured on a streptavidin pre-coated SA sensor chip (GE Healthcare). The analyte was either full-length GLI1 protein and/or GANT61. Purified recombinant (His)_6_-tagged ΔGLI-WT or ΔGLI-DM fragments were immobilized on a Ni-NTA chip [70]; the analyte was GANT61. Varying concentrations of GLI1 and/or GANT61, were passed over the DNA-biotin-SA, GLI1-CM5, or ΔGLI sensor chips at a flow rate of 20 μl/min for 3 min followed by 5 min dissociation in HBS-P buffer alone. Sensograms were recorded and the response units (RU) and maximum resonance units (Rmax) at equilibrium determined. All experiments were carried out on a Biacore Model 3000 and analyzed in bia evalution 4.0.1 (GE LifeSciences). The data were imported to Prizm to generate fit curves.

### GLI-luciferase Assay

The 12 GLI-binding site driven luciferase reporter (2 μg, GLI-luc, gift from Dr. Rune Toftgard, Karolinska Institutet [71]) and Renilla luciferase (0.2 μg, pRLTK) were cotransfected with either pBabe-Puro (2 μg, empty vector), full length GLI1 cDNA (2 μg, GLI1-WT), or full length GLI1-DM into HT29 cells using Lipofectamine 2000 (Invitrogen); 24 hr post-transfection, cells were treated with GANT61 (20 M) and allowed to grow for another 24 hr. Cells were subsequently harvested using the Dual luciferase reporter assay system (Promega Corporation) according to the manufacturer's protocol. Luciferase activity was detected by a Victor2 multilabel counter and normalized to Renilla luciferase activity as a control for transfection efficiency.

HT29-derived stable cell lines expressing the GLI-luciferase reporter were generated by transducing HT29 cells with pCignal Lenti-TRE-GLI-luciferase viral particles (SABiosciences) in the presence of polybrene (to a final concentration of 8 μg/ml) as flocculation agent to increase infectivity. The plate was gently swirled to mix. The cells were incubated overnight at 37°C in a humidified incubator with 5% CO_2_. Cells were tested after two passages for GLI-luciferase reporter activity. HT29-GLI-luc cells was transfected with either pBabe-Puro, GLI1-WT or GLI1-DM cDNA, and exposed to GANT61 (20 μM) for 24 hr. Live cell imaging was performed using a Bruker optical and X-ray small animal imaging system (Bruker Corporation) before or following treatment with GANT61.
